# Improved detection of magnetic interactions in proteins based on long-lived coherences

**DOI:** 10.1038/s42004-024-01195-2

**Published:** 2024-05-16

**Authors:** Octavian Ianc, Florin Teleanu, Andrei Ciumeică, Adonis Lupulescu, Aude Sadet, Paul R. Vasos

**Affiliations:** 1 Biophysics and Biomedical Applications Laboratory and Group, LGED, ELI-NP, “Horia Hulubei” National Institute for Physics and Nuclear Engineering IFIN-HH, 30 Reactorului Street, 077125 Bucharest-Măgurele, Romania; 2 Faculty of Physics, University of Bucharest, 405 Atomiștilor Street, 050663 Măgurele, Romania; 3 Interdisciplinary School of Doctoral Studies, University of Bucharest, 36-46 Mihail Kogălniceanu Bd, 050107 Bucharest, Romania; 4 Department of Chemistry, New York University, 100 Washington Square East, New York, NY 10003 USA; 5 Institute of Biological Chemistry, Faculty of Chemistry, University of Vienna, 1090 Vienna, Austria

**Keywords:** Solution-state NMR, Solution-state NMR, Solution-state NMR

## Abstract

Living systems rely on molecular building blocks with low structural symmetry. Therefore, constituent amino acids and nucleotides yield short-lived nuclear magnetic responses to electromagnetic radiation. Magnetic signals are at the basis of molecular imaging, structure determination and interaction studies. In solution state, as the molecular weight of analytes increases, coherences with long lifetimes are needed to yield advantageous through-space magnetisation transfers. Interactions between magnetic nuclei can only be detected provided the lifetimes of spin order are sufficient. In *J*-coupled pairs of nuclei, long-lived coherences (LLC’s) connect states with different spin-permutation symmetry. Here in, we show sustained LLC’s in protein Lysozyme, weighing 14.3 kDa, with lifetimes twice as long as those of classical magnetisation for the aliphatic protons of glycine residues. We found for the first time that, in a protein of significant molecular weight, LLC’s yield substantial through-space magnetisation transfers: spin-order transfer stemming from LLC’s overcame transfers from classical coherences by factors > 2. Furthermore, in agreement with theory, the permutation symmetry of LLC-based transfers allows mapping interacting atoms in the protein structure with respect to the molecular plane of glycine residues in a stereospecific manner. These findings can extend the scope of liquid-state high-resolution biomolecular spectroscopy.

## Abstract (web summary)

Solution NMR spectroscopy provides rich structural information on biomolecules, however, its resolution becomes limited when molecular size increases, due to short-lived nuclear magnetic responses to electromagnetic radiation. Here, the authors sustain long-lived coherences for the aliphatic protons of glycine residues within protein lysozyme, yielding substantial through-space magnetization transfers, and mapping interacting atoms in the protein structure.

## Introduction

As constituents of living matter evolved from primary elements, their structural symmetry decreased, thus enabling specific key-lock interactions. Unlike basic molecules such as H_2_, O_2_, N_2_, CO_2_, etc., few amino acids feature symmetry elements, and such elements are lost when amino acids are included in a protein fold. The loss of structural symmetry impacts the lifetime of nuclear-spin transitions: while interconversion between the nuclear-spin isomers of molecular hydrogen takes hours in solutions^1^, nuclear-spin transitions in liquid samples of amino acids have durations of seconds. This can be understood in terms of symmetry: due to the equivalence of the H_2_ protons, the intrapair dipolar interaction does not mix spin states belonging to different spin-permutation symmetries i.e., antisymmetric singlet-state para-hydrogen with symmetric triplet-state ortho-hydrogen^2^. For proton systems in an asymmetric environment, such as the aliphatic protons of an amino acid – with the notable exception of glycine - the chemical and magnetic equivalences between ^1^H spins are lost. Intra-spin-pair dipolar interactions become effective, reducing the lifetime of spin polarisation to several seconds. The most significant outcome of reduced magnetisation lifetimes is the limited intensity of magnetisation transfer via dipolar Overhauser effects^3,4^. For biomolecules in solution, intramolecular and inter-molecular magnetisation transfer is, to date, the main source for data on atomic interactions. Relaxation time constants and, consequently, magnetisation transfer for coherent spin superpositions, known as rotating-frame Overhauser effects^5^, are particularly sensitive to biomolecular size: they are scaled down as the effective molecular rotational correlation time in solution increases.

Solution structures of proteins can be solved by magnetic resonance spectroscopy in liquid state with fair resolution, up to a certain size. To date, liquid-state biomolecular structure elucidation for protein sizes beyond 40 kDa remain challenging^6,7^. Since protein fold and interactions in cells are essential for cell homeostasis and function, extending the reach of liquid-state NMR to proteins is crucial to rational drug design and mechanistic structural biology. Other liquid-state techniques based on chromophores can be difficult to use in molecular folds without perturbing the structure, therefore atomic resolution is mainly obtained non-invasively by NMR. Notable progress in surpassing the detection limit for increasing protein size has been made via the transverse relaxation optimized spectroscopy experiment (TROSY), relying on coupled heteronuclei that feature spin states with long lifetimes^8^. The lifetime enhancement in the TROSY experiment is due to the protection of heteronuclear spin states by the antagonistic action of correlated relaxation mechanisms (dipole-dipole and chemical shift anisotropy) of similar strength^6,9^. The current solution for structure determination for large proteins and biomolecular complexes relies on the dilution of strongly interacting proton spins in order to obtain the necessary transverse lifetimes for detection^7,10^. However, thinning of interacting spins in order to narrow spectral lines^6^ diminishes the number of obtainable structural constraints, as these stem from the very magnetic interactions that have been diluted^11^. Hydrogen spins are the first sensors of intramolecular and inter-molecular interactions. This study relies on detecting pairs of ^1^H spins with coherences that are only impacted to a small degree by the spin-spin dipolar interaction^12^. We show that a new approach for optimizing transverse ^1^H relaxation and improving Overhauser effects based on local molecular symmetry can become useful for the structural elucidation of large proteins and biomolecular complexes. Lately, computational methods are increasingly employed in protein structure determination. However, experimental data is especially needed in protein loops and at the frontiers of secondary structure regions, where glycine residues are often found. Glycine-based magnetic interactions are of importance to define mobile regions that are difficult to model correctly using AlphaFold^13^ or based on solid-state constraints derived from X-ray data. The structural flexibility of glycine-rich protein regions in the solution enables biological interactions; many partially disordered proteins are known to adopt biologically active conformations relying on their flexibility^14,15^.

Glycine residues have received increasing interest as singlet-state vessels for storing long-lived (hyper) polarization and monitoring kinetics^16–21^. For structural purposes, there remained the challenge to transfer the singlet-based spin order of *J*-coupled nuclei towards structural neighbours via Overhauser effects. It was discovered recently that magnetic resonance experiments can be conducted under conditions that establish local magnetic symmetry in two-spin systems, obtaining long-lived spin populations based on singlet states^22^ or long-lived transitions between the singlet and triplet states^23^. Introducing a magnetic field component to eclipse differences between local magnetic environments in aliphatic protons^24^, we have previously shown that local magnetic symmetry can be enhanced even at high fields, obtaining long-lived coherences (LLC’s)^12^. This opened the way for recording ^1^H magnetic resonance signals with enhanced resolution and relaxation time constants extended by up to a factor of 9 compared to classical coherences^25^ and was used in various experiments on small molecules^26–32^. The use of long-lived coherences for magnetisation transfer in a protein had not been achieved, though it was theoretically deemed possible^33^. We demonstrate now that ^1^H-based LLC’s with lifetimes twice as long as the lifetimes of standard transverse coherences, ${T}_{{LLC}} \, > \, 2{T}_{2}$ (*T*_1ρ_) can be observed in the 14.3 kDa protein Lysozyme. For the first time, LLC’s are used to transfer magnetisation from glycine residues to remote amino acids within the protein fold at high magnetic fields $({B}_{0}=22.3{T; } {\nu }_{H}^{0}=950{MHz})$. We prove via theory and experiments that the coherent superposition between singlet and triplet states within glycine residues of Lysozyme can act as long-lived magnetisation reservoirs capable of polarising nearby spins in a protein fold. This is a step forward in probing protein structure and interactions based on endogenous magnetic probes that can be detected non-invasively. Long-lived coherences can provide a way for assignment and structural elucidation of interactions while circumventing the need for isotopic enrichment. The narrow lines afforded by these coherences may add them to the set of NMR tools used for large proteins assignment provided that LLC detection in the direct dimension is achieved.

## Results and discussion

In the following discussion, we treat the magnetic interactions of Gly-H^α2,3^ proton magnetic moments denoted I and S, respectively. There are two possible orientations, $({\alpha ,\beta })_{I,S}$, for each of the two-proton spins with respect to an external magnetic field, ${B}_{0}$. In a free glycine molecule, methylene protons are chemically and magnetically equivalent, therefore the nuclear-spin eigenstates^34^ are: the spin-permutation-antisymmetric singlet state, ${S}_{0}=N(|{\alpha }_{I}{\beta }_{S}\rangle -|{\beta }_{I}{\alpha }_{S}\rangle )$ and the three symmetric triplet states, ${T}_{+1}=|{\alpha }_{I}{\alpha }_{S}\rangle ,{T}_{0}=N(|{\alpha }_{I}{\beta }_{S}\rangle +|{\beta }_{I}{\alpha }_{S}\rangle ),{T}_{-1}=|{\beta }_{I}{\beta }_{S}\rangle$, with $N={2}^{-1/2}$. The decays of singlet-state-based populations and transitions are the least perturbed by spin-permutation symmetric interactions, such as the dipole-dipole relaxation between the two nuclei that cannot mix states with different symmetries.

### Long-lived coherences

**Fig. 1:**
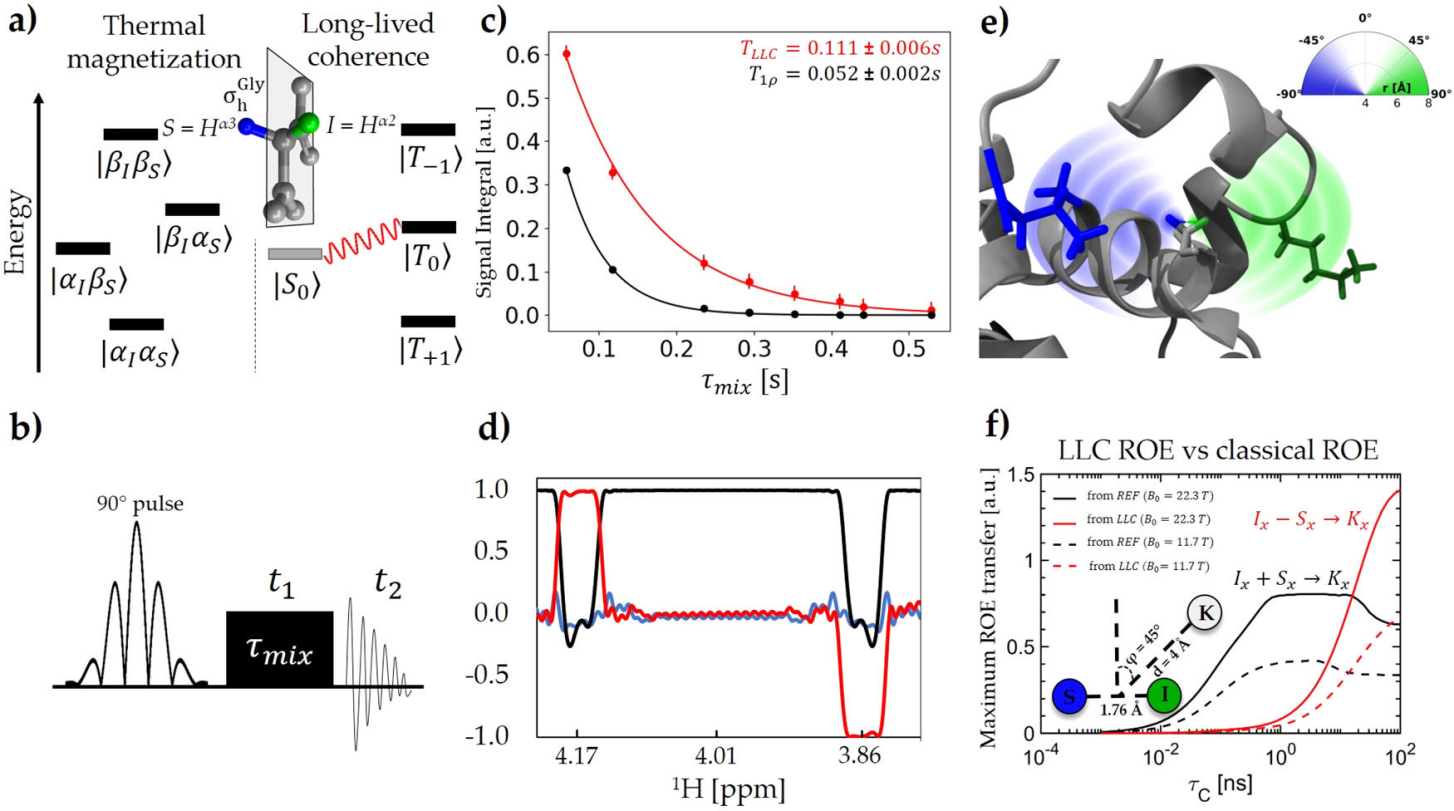
Method for the detection of LLC-based transfer in proteins. **a** Energy level diagram of spin states characteristic to a *J*-coupled two-spin-1/2 system. Thermal magnetisation is a population imbalance between the outer states, while long-lived coherences are superpositions between the singlet state and the central triplet states. Inset: glycine backbone highlighting the H^α2,3^ protons denoted *(I,S)* and the mirror plane ${\sigma }_{h}^{{Gly}}$ which interconverts the two spins. **b** The 1D/2D pulse sequences used to excite multiple narrow bands of the protein’s proton spectrum centred around the targeted Gly-H^α^ resonances and sustain LLC’s during transfer. The initial phase offset between the two components of the 90° dual-frequency pulse can be adjusted to generate either long-lived coherences (‘LLC’ experiment), or classical transverse coherence (reference ‘REF’ experiment). (see “Materials and Methods” section). **c** Signal decay of the LLC versus transverse magnetisation during the spin-lock period (${\tau }_{{mix}}$) measured at $1/{J}_{{IS}}^{{Gly}}$ intervals for Gly117 residue. **d** Excitation profile of the 90° dual-frequency pulse used to excite LLCs (red) and standard transverse coherences (black, shifted). Residual z-axis magnetisation is shown in blue. The y-axis shows normalised magnetisation excited for each nuclear spin. **e** Graphic representation of the LLC-ROE magnetisation transfer in a sphere surrounding the two glycine protons H^α2^ (green) and H^α3^ (blue) highlighting the stereospecific interaction of neighbouring protons. Depending on relative distances to the two (I,S) methylene protons, surrounding spins K will display either negative (when they are found in the space region closer to H^α2^, outlined in green) or positive ROE build-up curves (when they are found in the space region closer to H^α3^, outlined in blue). **f** Numerical simulations (notebook provided in Supplementary Note 3) highlighting the dependence of maximum expected transfer towards a third spin K via rotating-frame Overhauser effects (ROE), starting from ${\rho }_{{LLC}}^{0}={I}_{x}-{S}_{x}$ (red) compared to ${\rho }_{{REF}}^{0}={I}_{x}+{S}_{x}$ (black), the rotational correlation time increases. The transfer from LLC gradually increases and surpasses the classical ROE transfer. Structural parameters of the three-spin-1/2 system are provided as an inset.

In the singlet-triplet basis, long-lived coherences can be expressed as superposition between the singlet and the central triplet state (Fig. 1a):

1 $${Q}_{{LLC}}=|{S}_{0}\rangle \langle {T}_{0}{|}+|{T}_{0}\rangle \langle {S}_{0}{|}+i(|{S}_{0}\rangle \langle {T}_{0}{|}-|{T}_{0}\rangle \langle {S}_{0}|)$$

The expression of LLC’s in terms of individual coherences at the two aliphatic proton sites of Gly residues can readily be recognised when LLC’s are expressed as Cartesian operators^12,35^: as detailed in Supplementary Note 2, they have a real component ${Q}_{{LLC}}^{//}={I}_{x}-{S}_{x}$ and an imaginary component ${Q}_{{LLC}}^{\perp }=(2{I}_{z}{S}_{y}-2{I}_{y}{S}_{z})$. LLC’s can be initialized as ${\rho }_{{LLC}}^{0}={I}_{x}-{S}_{x}$, but this state is inaccessible in the magnetic-equivalence regime of free glycine and can only be excited and detected when a symmetry-breaking mechanism is present^29^. When glycine residues are in a peptide or protein fold, the two methylene protons are chemically and magnetically inequivalent. This allows the manipulation of individual spins via selective pulses and the initialization of LLC’s. However, magnetic relaxation is fast-paced in this situation. The mirror plane symmetry element ${\sigma }_{h}^{{Gly}}$ (Fig. 1a) can be effectively reintroduced by applying a high-amplitude radio-frequency magnetic field at the average value of the Larmor frequencies of the two spins. In practice, we exploit the ability to shift between magnetic regimes: after exciting LLC’s by spin-selective pulses granted the different chemical shifts, we sustain them in a magnetic-equivalence setting using continuous-wave (CW) irradiation. Experimentally, LLC’s are excited via the permutation-antisymmetric term ${\rho }_{{LLC}}^{0}={I}_{x}-{S}_{x}$ and their coherent evolution during the mixing time τ_mix_, when they are sustained by CW irradiation, is described by:

2 $${\rho }_{{LLC}}\left({\tau }_{{mix}}\right)=\left({I}_{x}-{S}_{x}\right)cos \left(2\pi {J}_{{IS}}{\tau }_{{mix}}\right)+\left(2{I}_{z}{S}_{y}-2{I}_{y}{S}_{z}\right)sin \left(2\pi {J}_{{IS}}{\tau }_{{mix}}\right)$$

where *J*_*IS*_ is the scalar coupling constant between the two methylene protons in the glycine moiety. LLC’s have an oscillatory evolution $({\nu }_{{LLC}}={J}_{{IS}})$ and the recorded 1D spectra consist of a superposition of in-phase and anti-phase peaks depending on the mixing period ${\tau }_{{mix}}$. The oscillating transfer from glycine-based LLC’s to neighbouring spins with a frequency equal to the scalar coupling (${\nu }_{{LLC}}={J}_{{IS}}$) is dampened by relaxation as molecular tumbling slows down^33^. Calculations indicate that oscillations of observed signals stemming from the magnetisation source are quenched both by relaxation and by small oscillations at the transfer site K, induced by local couplings (see Supplementary Note 3 and Figs. S3, S4). Local anisotropic motions, cross-correlated effects and spin diffusion are effects that go beyond the simple case of three-spin systems that contribute to the observed transfer curves.

In order to follow individual glycine residues in a large protein, we developed a new technique to excite ${\rho }_{{LLC}}^{0}={I}_{x}-{S}_{x}$ term using a 90° dual-frequency pulse^36^ suited for protein experiments due to its small excitation windows. The excitation pulse is adapted for each glycine residue in Lysozyme based on the chemical shift values of the individual proton spins (See Supplementary Note 1 Table S1) which were measured with a workflow based on selective long-lived state filters^17,37^. The LLC excitation and the extended lifetimes were probed using the 1D / 2D pulse sequence in Fig. 1b. For comparison, the dual-frequency pulse was adapted to excite classical transverse magnetisation ${\rho }_{{REF}}^{0}={I}_{x}+{S}_{x}$ and its decay was measured to be up to 2 times faster than LLC’s for Gly residues in Lysozyme (Fig. 1c illustrates the *T*_LLC_ fitting in Gly117 and Fig. 1d shows excitation profile of the 90° dual-frequency pulse used to excite LLCs and standard transverse coherences). A characteristic spectral feature of LLC experiments are the cross-peaks in the 2D Fourier-transformed spectrum obtained with the pulse sequence in Fig. 1b where the mixing time is used as a pseudo-indirect dimension. Due to the coherent evolution during CW irradiation (Eq. 2), symmetric cross-peaks in the indirect dimension F_1_ appear at a position ${J}_{{IS}}^{{Gly}117}\approx 17.5{Hz}$ from the baseline at the Gly117 resonances (See Supplementary Note 4 Fig. S6). These cross-peaks are absent in the 2D REF experiment, as expected. LLC-ROE spectroscopy is a fast way of probing dipolar interactions of LLC’s with external relaxation sources like neighbouring spins (Fig. 1e) in the environment of the two glycine aliphatic protons.

### Magnetisation transfer via LLC-ROE experiments

The sensitivity of LLC’s to the presence of nearby nuclei yields new information compared to classical coherences. We have previously described the interaction of a singlet-triplet superposition with a neighbouring spin via rotating-frame Overhauser effect (ROE) both theoretically and via experiments for small-molecule systems^33^. Based on the derived equations, the theoretical dependence of the maximum magnetisation transfer from LLC to a neighbouring spin (See Supplementary Note 2) on the rotational correlation time modulating the dipolar interaction, ${\tau }_{C}$, is compared to the classical transfer from transverse magnetisation in Fig. 1f, Supplementary Note 3 Fig. S5. LLC’s feature a significant advantage in terms of transfer yields compared to standard coherences as the rotational correlation time increases beyond 10 ns. Moreover, the difference between LLC-based and standard Overhauser transfer increases with the static magnetic field, *B*_0_, at which the experiment is performed. At a magnetic field corresponding to proton Larmor frequency of 950 MHz, proteins of the molecular weight of Lysozyme can be expected to feature LLC-based transfer comparable to or higher than the classical transverse transfer, depending on local motions.

**Fig. 2:**
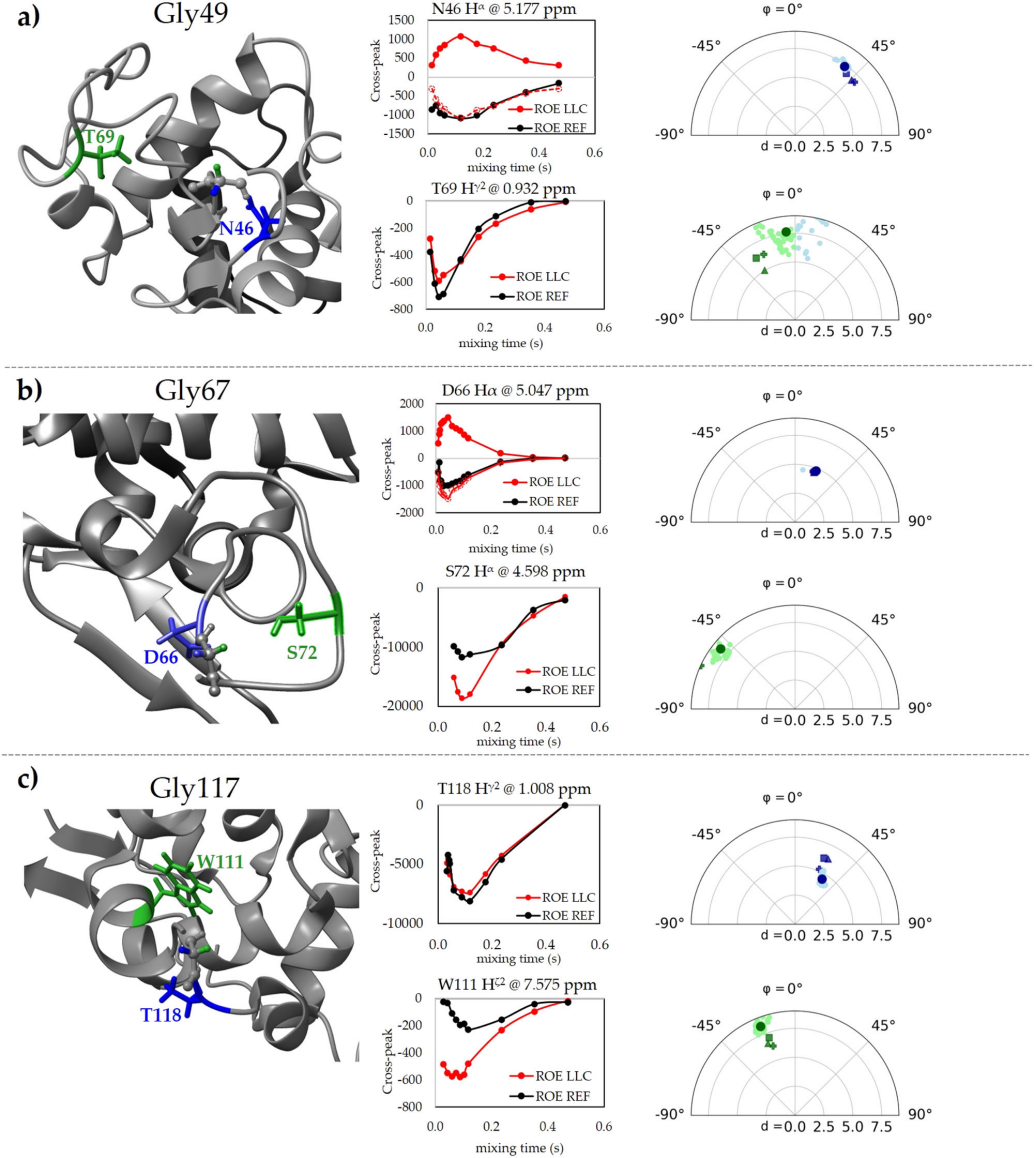
‘LLC-ROE’ (red) versus reference ‘REF’ ROE (black). Build-up data recorded using the pulse sequence shown in Fig. 1b for several cross-peaks of Gly49 (**a**), Gly67 (**b**) and Gly117 (**c**). Gly-H^α2,3^ protons are highlighted in green and blue, respectively, and residues to which Overhauser transfer is detected are rendered using the same spatial colouring. The change in sign of the LLC-ROE build-up matches the spatial proximity of the neighbouring atoms relative to the glycine molecular plane ${\sigma }_{h}^{{Gly}}$, i.e., atoms closer to H^α2^ (green) have negative build-ups, while the ones closer to H^α3^ (blue) have positive sign. For LLC-ROEs with build-up signs opposed to classical ones, LLC-ROE data points multiplied by −1 (dotted red line) are shown in order to better evaluate the magnitude of transferred LLC magnetisation compared to classical ROEs. Polar plots are given for neighbouring atoms positions around the Gly aliphatic protons: the radius represents the distance between the specified ‘K_i_’ neighbour nuclei and the middle point of the IS segment; the polar angle is the angle between the median to IS and the distance vector relying spin K to the middle point of the IS segment (as shown in Fig. 1e, f and Supplementary Note 3). The φ angles and distances in X-ray structures ‘1DPX’, ‘4XJF’ and ‘9LYZ’ are represented using triangles, squares and plus signs, respectively. The structural data for the angle and distance values derived from the 50 NMR-derived coordinate sets (‘1E8L’ PDB entry) are denoted by small circles, and their average is represented by a large circle.

Magnetisation transfer from ${\rho }_{{LLC}}^{0}={I}_{x}-{S}_{x}$ to the adjacent nuclei K_i_ occurs with different signs when K is close to either I or S, due to the fact that ${\rho }_{{LLC}}^{0}$ is permutation antisymmetric. Figure 1e shows expected regions in space outlined in green for transfer expected to occur via LLC-ROE with the same sign as in classical ROE and in blue when transfer is expected with the opposite sign (See Supplementary Note 3.1 Fig. S2 for the angular dependence of LLC-ROE transfer). Experimentally, using the pulse sequence in Fig. 1b, neighbour K spin signal intensities in LLC-ROE experiments starting from ${\rho }_{{LLC}}^{0}={I}_{x}-{S}_{x}$ were compared with REF-ROE experiments starting from ${\rho }_{{REF}}^{0}={I}_{x}+{S}_{x}$ as a function of the mixing time ${\tau }_{{mix}}$. The results for Gly49, Gly67, and Gly117 residues, which yield the best-resolved signals, are provided in Fig. 2. For each glycine residue, relative distances to Gly-H^α2^ and Gly-H^α3^ protons were confirmed using the relative intensities of 1D horizontal slices from a standard 2D ROESY spectrum taken at the indirect-dimension frequencies of Gly-H^α2^ and Gly-H^α3^ frequencies. As predicted by the LLC theory, the cross-peak build-up sign was experimentally observed to change in LLC-ROE with respect to REF-ROE for neighbouring interaction sites closer to glycine’s proton *S* (Gly-H^α3^) than to glycine’s proton *I* (Gly-H^α2^). For instance, the LLC-ROE signs of Gly69 neighbour D66-H^α^ and Gly49 neighbour N46-H^α^ are opposed to the reference ROE signs. The opposite signs for ROE-LLC build-ups stemming from neighbours in close proximity to Gly-H^α2^ compared to ROE-LLC from neighbours of Gly-H^α3^ provide an easy route for deriving new biomolecular structure constraints or for refining existing structures. LLC-ROE data can be used for structure validation, especially in mobile loop regions where glycine residues abound.

We have verified the consistency of the sign dependency of ROE build-ups with both liquid-state NMR (‘1E8L’ PDB entry^38^) and X-ray structures of Lysozyme (‘1DPX’, ‘4XJF’, and ‘9LYZ’ PDB entries^39–41^). This analysis confirmed the spatial proximity of identified neighbour nuclei with respect to the glycine molecular plane ${\sigma }_{h}^{{Gly}}$ (Fig. 2). In cases where there is an important degree of dispersion between existing structural experimental data from the literature, LLC-ROE experiments can help choose the correct conformation – in the case of Gly49-Hα2,3 interaction with the Thr69 methyl, the reciprocal position reflected in the '1DPX' crystal structure are confirmed by LLC-ROE. Other structures with local conformations confirmed by LLC-ROE data are ‘4XJF’, ‘9LYS’, and 5 of the available 50 NMR-based models in '1E8L'. The sign of LLC-ROE derived φ angles agreed with the existing structural data, and in the case of the Gly69-Ser72H^α^ interaction, a larger φ angle measured in both X-ray and NMR structures correlates with higher LLC-ROE intensity, as predicted by theory. The only instance where obtained LLC-ROE data were found to be at variance with respect to existing structural data was the position of T118 methyl with respect to Gly117. In this case, further structural refinement based on LLC-ROE would change the φ angle by ca 20 degrees. Importantly, for several of the reported interactions in Fig. 2, the LLC-ROE reference intensity is clearly stronger than the classical reference ROE intensity, by a factor 2 for Ser72-H^α^ interaction with Gly67 aliphatic protons and by almost a factor 3 for the Trp111-H^ζ2^ interaction with Gly117 aliphatic protons. The Asp66-H^α^ interaction with Gly67 aliphatic protons is also more intense in LLC-ROE than in classical ROE - with opposed sign, as predicted by the structure. The intensities of the various local interactions in ROE-LLC and classical ROE depend on the local effective anisotropic correlation times for molecular motions as much as on the angles and distances. This is the first experimental proof of the emergence of several more intense LLC-ROE signals compared to classical ROE signals in a protein of significant size in solution. These features render the newly-proposed magnetisation transfer method useful for probing the magnetic interactions of proteins using proton spectroscopy (the interaction of Lysozyme with its biological target is shown in Fig. S7 of Supplementary Note 5).

In conclusion, we propose a new method to detect magnetization transfer from long-lived coherences in glycine residues to neighbouring atoms. We show for the first time that, in a protein of molecular weight beyond 14 kDa, rotating-frame Overhauser effects based on long-lived coherences can be more effective than transfer from standard coherences. This finding agrees with predictions by theory and numerical simulations that LLC-ROE transfer should be enhanced compared to standard ROE in biomolecules featuring effective rotational correlation times close to 10 ns or above. Detection at increased static magnetic field strengths *B*_0_ further improves LLC-ROEs. Stereospecific structural constraints are derived from LLC-ROE, as long-lived coherences yield build-ups with different signs for interactions with ^1^H neighbours that are close to Gly-H^α2^ or Gly-H^α3^, respectively. LLC-ROE is to be considered for deriving atomic-resolution structural constraints in the case of biomolecular complexes in solution.

## Materials and methods

The Lysozyme protein (29 mg, MW = 14300 g mol^−1^) with natural-abundance spin isotopes was dissolved in D_2_O (0.55 ml) at pH = 4 as described in ref. ^44^. NMR spectra were recorded at T = 310 K on a Bruker Avance spectrometer operating at B_0_ = 22.3 T (proton Larmor frequency *ν*_0_(^1^H) = 950 MHz) equipped with a cryogenically-cooled probe head optimised for ^1^H sensitivity (‘triple-channel inverse probe’-‘TCI’). Proton reference and 1D LLC experiments were recorded with 8 transients and a recovery delay of 8 s. The Gly-(H^α2^, H^α3^) pairs in Lysozyme feature *J*_IS_ coupling values *J*_IS_~17 Hz and their respective frequency differences Δν_IS_ at the given B_0_ value are provided in Table S1 (See Supplementary Note 1 Fig. S1). To record selective LLC and REF experiments, we adapted the pulse sequence described in Fig. 1b for each glycine proton system. The carrier frequency of the 90° dual-frequency E-BURP2 pulse was placed on the most downfield proton and had a duration of 50 ms with an amplitude of 80 Hz. The initial phase offset between the two components of the 90° dual-frequency pulse can be adjusted to generate either long-lived coherences (‘LLC’ experiment) or classical transverse coherence (reference ‘REF’ experiment). The excitation range of the selective excitation pulse was ±30 Hz around each of the Gly resonances (Fig. 1d). The excitation windows at the frequencies of I and S spins are centred on the two Gly-Hα resonances. The initial phase offset between the two components of the dual-frequency pulse was varied in order to excite either ${\rho }_{{LLC}}^{0}={I}_{x}-{S}_{x}$ (LLC experiments) or ${\rho }_{{REF}}^{0}={I}_{x}+{S}_{x}$ (REF experiments). For the ‘REF’ experiments, both the transverse components of I and S spins are excited with the same sign by adapting the initial phase offset. The CW-sustained mixing time is used to create the indirect dimension in 2D experiments. The acquired number of points in the indirect and respectively direct dimensions was F_1_ = 64 and F_2_ = 2048. The indirect-dimension increment was 1  ms. 2D LLC spectra were recorded summing ns = 32 transients. The continuous-wave irradiation pulse was set in the middle of the two Gly-H^α^ resonances with an amplitude of 4 kHz. Experimental data was processed using TopSpin 4.2, MestreNova v15.0.1, CCPN^43^ v3.2.0, NMRglue^44^ v0.1, and SPARKY^45^ v3.1.5. Spin dynamics calculations were performed using SpinDynamica^46^ v3.7.1 and Spinach^42^ 2.7.6049 libraries within a four-spin system (featuring spins *I* and *S*, neighbouring spin *K*, and an additional spin R close to *K*, with the only *J*-coupling constant active between the two Gly aliphatic protons I and S).

### Supplementary information

Supplementary Information

## Data Availability

Data acquired during this study is available at https://ncloud.eli-np.ro/index.php/s/kMxNlQBgfPYc1RT and we will answer any requests for additional information.

## Code availability

Software codes used for calculations are given in Supplementary Note 3.2.
