# Supplementary material for: Improved detection of magnetic interactions in proteins based on long-lived coherences
Source: Commun Chem. 2024 May 16;7:112. doi: 10.1038/s42004-024-01195-2 (PMC11099074; doi:10.1038/s42004-024-01195-2)
Supplement: Supplementary file 1 — Supplementary Information [file 42004_2024_1195_MOESM1_ESM.pdf]

Supporting Information

for

Improved detection of magnetic interactions in proteins based on long-lived coherences

Table of Contents

1. **Supplementary Note 1:** Experimental workflow for selective long-lived coherences experiments in large proteins
2. **Supplementary Note 2:** Theoretical analysis of rotating-frame Overhauser transfer from long-lived states and coherences
3. **Supplementary Note 3:** Calculations of ROE-LLC effects within model spin systems

   3.1 **Supplementary Note 3.1:** Angular dependence of ROE-LLC

   3.2 **Supplementary Note 3.2:** Matlab notebooks for calculation of LLC-ROE intensities

   3.3 **Supplementary Note 3.3:** Build-up in NOE vs ROE and ROE_LLC spectroscopy
4. **Supplementary Note 4:** 2D ROE LLC experiments and spatial neighbor assignments
5. **Supplementary Note 5:** Lysozyme interactions with trisaccharide NAG-NAM-NAG

1. **Supplementary Note 1:** Experimental workflow for selective long-lived coherence experiments in large proteins

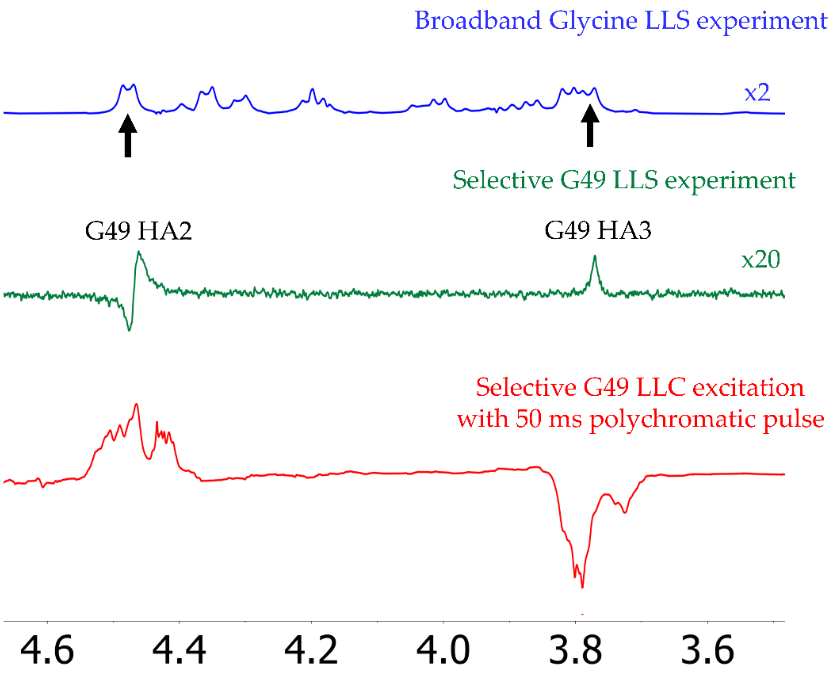

Figure S1. Workflow employed for the identification of glycine resonances and selective LLC excitation of glycine residues in Lysozyme at 950 MHz. Step 1: Broadband excitation and detection of LLS via pulse sequence of Ref. [3]. Glycine resonances are identified (blue) in this way, but are not clearly assigned. Step 2: Based on knowledge of resonance frequencies acquired at Step 1, selective excitation and detection of Gly49 signal (green –LLS-relaxation filtered) was detected using the frequency-selective pulse sequence of Ref. [4]. This method allows for unambiguous assignment of coupled spins resonances in each glycine using an LLS filter. Step 3. Once both resonances of one glycine residue are assigned, a 50 ms polychromatic EBURP2 pulse (with initial frequency offset) allows simultaneous excitation of transverse magnetization in two spectral regions with opposite sign, leading to LLC excitation of the selected Glycine residue (red). The lower spectrum shows the experimental profile of the selective pulse in the protein sample. The excitation spectral regions (with a spectral width around 70 Hz each) are centered on the previously assigned glycine resonances.

Table S1. Assigned glycine resonances using the workflow method described above

**Residue**	**HA2 (ppm)**	**HA3 (ppm)**	**Δν (Hz) @ 950 MHz**
G4	4.384	4.034	332.5
G16	4.177	3.971	195.7
G49	4.471	3.773	663.1
G67	4.193	3.887	290.7
G117	4.185	3.860	308.8
G126	4.351	3.806	517.8

2. **Supplementary Note 2:** Theoretical analysis of rotating-frame Overhauser transfer from long-lived states and coherences

In the following we consider the evolution of a system of three spins, *I*, *S*, and *K*, with relaxation due to dipolar interactions only, and *J*-coupling between *I* and *S*. For Overhauser magnetization transfer starting from long-lives states, we note that the cross-relaxation rate constant between $\rho_{LLS} = \hat{\mathbf{I}} \cdot \hat{\mathbf{S}}$ and another 1-spin order term of the third spin $\widehat{K}_{\mu}$, with $\mu = x, y, z$, is null ($\sigma_{LLS/K_{\mu}} = 0$) when only dipolar interactions are considered. This can be understood in terms of operators' mathematical properties as follows: because dipolar interactions are characterized by 2-spin order Hamiltonians $\widehat{H}_{DD} = b_{dd}(3\hat{A}_z\hat{B}_z - \widehat{\mathbf{A}} \cdot \widehat{\mathbf{B}})$, the double-commutation superoperator $\hat{\hat{\Gamma}}_{DD} = [\![\widehat{H}_{DD}, [\widehat{H}_{DD}, \quad]\!]$ can only modify the correlation order of a targeted operator with 0 or $\pm$ 2, as discussed more thoroughly in Ref. [1]. Thus, the singlet population $\rho_{LLS} = \hat{\mathbf{I}} \cdot \hat{\mathbf{S}}$ cannot be converted directly through Overhauser effect into a 1-spin term such as polarization at a third neighboring spin. Nonetheless, if cross-correlated effects between the dipolar mechanism and other relaxation interactions characterized by 1-spin order Hamiltonians are present, the cross-relaxation rate $\sigma_{LLS/K_{\mu}}$ will become non-null and polarization transfer can take place. Chemical shift anisotropy (CSA) is such a mechanism that should allow polarization transfer from long-lived states based on heavier ½-spin nuclei, such as ^{13}C, ^{19}F and ^{31}P, where the shielding anisotropy is large. Additional analytical investigation was performed within SpinDynamica [2].

For the case of long-lived coherences (LLC's), discussed in the main text:

$$Q_{LLC} = |S_0\rangle\langle T_0| + |T_0\rangle\langle S_0| + i(|S_0\rangle\langle T_0| - |T_0\rangle\langle S_0|), \tag{1}$$

The expression of long-lived coherences in terms of Cartesian component can be expressed, in the habitual z-axis quantization given by an external B_0 field, as a real component $Q^{//}_{LLC\ (z-axis)} = I_z - S_z$ and one imaginary component with zero-quantum terms, $Q^{\perp}_{LLC(z-axis)} = (2I_xS_y - 2I_yS_x)$. In the presence of a continuous-wave sustaining field B_1 applied along the x-axis of the laboratory frame, with amplitude superior to the frequency difference between the two spins, the quantization axis changes from the z to the x axis, yielding: $Q^{//}_{LLC} = I_x - S_x$ an, $Q^{\perp}_{LLC} = (2I_zS_y - 2I_yS_z)$. Their time evolution is given by:

$$\rho_{LLC}(\tau_{mix}) = (I_x - S_x)cos(2\pi J_{IS}\tau_{mix}) + (2I_zS_y - 2I_yS_z)sin(2\pi J_{IS}\tau_{mix}), \tag{2}$$

The operator $Q^{x}_{LLC} = \hat{I}_x - \hat{S}_x$ is a linear combination of 1-spin order terms and has a non-zero cross-relaxation rate constant with the third spin operator $\widehat{K}_x$ equal to the difference between individual cross-relaxation rates of the two I and S spins with the third spin K ($\sigma_{\rho^{//}_{LLC}/K_x} = \sigma_{I_x/K_x} - \sigma_{S_x/K_x}$). This leads to a pronounced angular dependence of the LLC-ROE transfer with a maximum cross-relaxation rate when the three spins are collinear, and a minimum value when the third spin K is on the perpendicular bisector of the segment connecting I and S nuclei. The

operator $Q_{LLC}^{yz} = 2\hat{I}_z\hat{S}_y - 2\hat{I}_y\hat{S}_z$ is a sum of 2-spin order terms and, based on the above discussion, has a null cross-relaxation rate with any form of polarization on the third spin $\widehat{K}_\mu$, with $\mu = x, y, z$ ($\sigma_{\rho_{LLC}^{\perp}/K_x} = 0$). Because long-lived coherences oscillate between the two components (Q_{LLC}^{x} and Q_{LLC}^{yz}) with a frequency equal to the scalar coupling constant J_{IS}, the ROE transfer towards the $\widehat{K}_X$ obtained from spin-order initially excited via Q_{LLC}^{x} will oscillate with the same frequency, but with a phase difference of $\pi/2$ with respect to the LLC's evolution.

With increasing molecular weight or solvent viscosity, the characteristic rotational correlation times of analyte molecules, τ_C, increase. LLC's lifetimes become shorter than one oscillation period and the ROE transfer is described by a non-oscillating bi-exponential curve. Nonetheless, due to the longer lifetime of LLC compared to classical coherences (up to 9 times higher) [3], an enhanced ROE transfer is predicted by numerical simulations for large proteins [4].

To simplify notation, we introduce for the expectation values of coherences involved in the ROE process the following symbols: $K = \langle K_x \rangle$, $I = \langle I_x \rangle$, $S = \langle S_x \rangle$, $Q_+ = \langle I_x + S_x \rangle$, $Q_{LLC}^{x} = \langle I_x - S_x \rangle$, and $Q_{LLC}^{yz} = \langle 2I_yS_z - 2I_zS_y \rangle$. Relaxation in the *I-S-K* system is described by auto-relaxation rates $\rho_I = \rho_S, \rho_K$ and cross-relaxation rates $\sigma_{IS}, \sigma_{IK}, \sigma_{SK}$. Taking into account coherent evolution due to the *J*-coupling between the *I* and *S* spins, it is found that

$$dI/dt = -\pi J Q_{LLC}^{yz} - \rho_I I - \sigma_{IS} S - \sigma_{IK} K, \qquad [3]$$

$$dS/dt = +\pi J Q_{LLC}^{yz} - \rho_S S - \sigma_{IS} I - \sigma_{IK} K, \qquad [4]$$

$$dQ_{LLC}^{yz}/dt = 2\pi J Q_{LLC}^{x} - (\rho_I - \sigma_{IS}) Q_{LLC}^{yz}.$$

From Eq. [1-3], the evolution of Q_{LLC}^{x} and $Q_{LLC}^{yz}$is given by

$$dQ_{LLC}^{x}/dt = -2\pi J Q_{LLC}^{yz} - \rho_{LLC} Q_{LLC}^{x} - \sigma_{LLC}^{K} K \qquad [5]$$

$$dQ_{LLC}^{yz}/dt = +2\pi J Q_{LLC}^{x} - \rho_{LLC} Q_{LLC}^{yz} \qquad [6]$$

where $\rho_{LLC} = \rho_I - \sigma_{IS}$ and $\sigma_{LLC}^{K} = \sigma_{IK} - \sigma_{SK} = \sigma_-$. The equation for the change rate of K,

$$dK/dt = -\rho_K K - \sigma_{IK} I - \sigma_{SK} S, \qquad [7]$$

can be recast as

$$dK/dt = -\rho_K K - \tfrac{1}{2}\sigma_{LLC}^{K} Q_{LLC}^{x} - \tfrac{1}{2}\sigma_+ Q_+, \qquad [8]$$

where $\sigma_+ = \sigma_{IK} + \sigma_{SK}$. Because of the appearance of Q_+ in Eq. [7], an additional equation must be added which, according to Eq. [1] and [2], is

$$dQ_+/dt = -\rho_+ Q_+ - \sigma_+ K, \qquad [9]$$

where $\rho_+ = \rho_I + \sigma_{IS}$. However, according to the experimental procedure for producing LLC, initially $Q_+(0) = \langle I_x + S_x \rangle(0) = 0$ and it can be expected that $Q_+(t)$ remains small throughout the ROESY irradiation. Therefore, we can ignore Eq. [8] and neglect $\sigma_+ Q_+$ term in Eq. [7], such that we are left with the system of equations

$$dQ^x_{LLC}/dt = -2\pi J Q^{yz}_{LLC} - \rho_{LLC} Q^x_{LLC} - \sigma^K_{LLC} K, \quad [10]$$

$$dQ^{yz}_{LLC}/dt = +2\pi J Q^x_{LLC} - \rho_{LLC} Q^{yz}_{LLC}, \quad [11]$$

$$dK/dt = -\rho_K K - \tfrac{1}{2}\sigma^K_{LLC} Q^x_{LLC}. \quad [12]$$

A simple approximate analytical solution can be obtained by assuming that, starting with the initial condition $\rho(0) = Q^x_{LLC}$, the influence of K ($K(0) = 0$) on the time evolution of Q^x_{LLC} and Q^{yz}_{LLC} can be neglected. This is sensible as long as $K(t)/Q^x_{LLC}(0)$ is small throughout of ROESY irradiation. Neglecting K in Eq. [9,10] we find the solution for Q^x_{LLC} as

$$Q^x_{LLC}(t) = \exp(-\rho_{LLC} t)\cos(2\pi J t). \quad [13]$$

Accordingly, Eq. [11] becomes an inhomogeneous differential equation with 'source term' $-1/2\,\sigma^K_{LLC} Q^x_{LLC}(t)$, where $Q^x_{LLC}(t)$ is given by Eq. [12]. The solution of the inhomogeneous differential equation is

$$K(t) = Ae^{-\rho_K t} - e^{-\rho_{LLC} t}[A\cos(2\pi J t) + B\sin(2\pi J t)], \quad [14]$$

where

$$A = \frac{(\rho_K - \rho_{LLC})\sigma^K_{LLC}}{2\sqrt{(\rho_K - \rho_{LLC})^2 + 4\pi^2 J^2}} \quad B = \frac{2\pi J \sigma^K_{LLC}}{2\sqrt{(\rho_K - \rho_{LLC})^2 + 4\pi^2 J^2}}$$

The accuracy of Eq. [14] was tested for various values of the relevant parameters. We have found that Eq. [14] is accurate for realistic values of ρ_{LLC}, ρ_K, and σ^K_{LLC}. Significant deviations between $K(t)$ and numerically-computed values occur only when σ^K_{LLC} is comparable to ρ_K (unlikely, as internuclear distances $r_{IK}\ and\ r_{SK}$ are in all cases larger than r_{IS}).

3. **Supplementary Note 3:** Calculations of ROE-LLC effects within model spin systems

3.1 **Supplementary Note 3.1: Angular dependence of ROE-LLC**

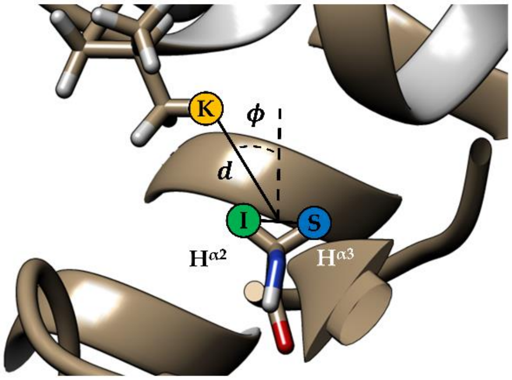

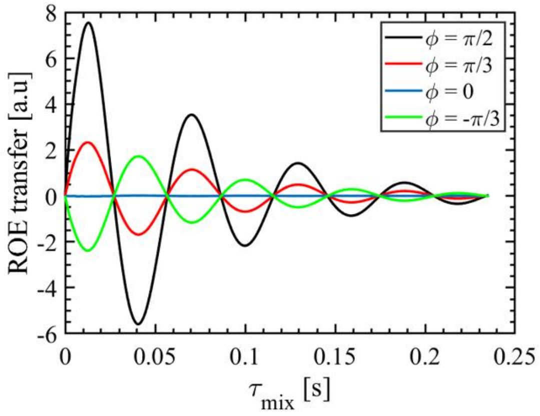

Figure S2. Simulated angular dependence of the magnetization transfer from ROE LLC as function of ϕ angle at $\tau_C = 15\,ns$ rotational correlation time. The $\{I, S, K\}$ spin system has the following geometrical constrains: the internuclear distance between I and S spins is 1.76 Å and the distance between K spin and the middle point of the I-S segment is d = 4.0 Å (Spinach [7] notebook provided as supplementary materials). Stereospecific ROE transfer is obtained from LLC's by sign reversal of the build-up due to a closer proximity of the K spin toward either I or S spins.

Predicted oscillations at the source frequency (Gly-$^2J_{IS}$ = 17 Hz) in the LLC-transferred magnetization can hamper the magnetization build-up, but are in practice quenched over large domains of the observed times by oscillations of the detected spins K, explaining the experimentally-detected rotating-frame Overhauser transfers.

3.2 Supplementary Note 3.2: Matlab notebooks for calculation of LLC-ROE intensities

Rotating-frame Overhauser transfer intensities in presence of additional external spins

```
figure ();
%System's parameters
time_array = 0.1:0.001:0.19;
rhoLLC = 0.2;
JN = 2;
sigma = 5;
rhoK = 8;
J = 17;

DR = 2 * sqrt((rhoK - rhoLLC) .^ 2 + (2 * pi * J) .^ 2); %Denominator for the values
of A and B

A = (rhoK - rhoLLC) .* sigma ./ DR;
B = 2 * pi * J * sigma ./ DR;
```

```
roe = A .* exp(-rhoK .* time_array) - exp(-rhoLLC * time_array) .* (A .* cos(2 * pi *
J * time_array) + B .* sin(2 * pi * J * time_array)); % Compute the non-oscillating
ROE transfer
roe_osc = -roe .* cos(2 * pi * JN * time_array); % Add the small-frequency oscillation

% Plot the two magnetization evolutions
plot(time_array, roe, '-g');
hold on;
plot(time_array, roe_osc, '-b');
xlabel("t [s]")
ylabel("Amplitude [a.u.]")
%legend({'J_{N} = 0', 'J_{N} = 2 Hz'})
```

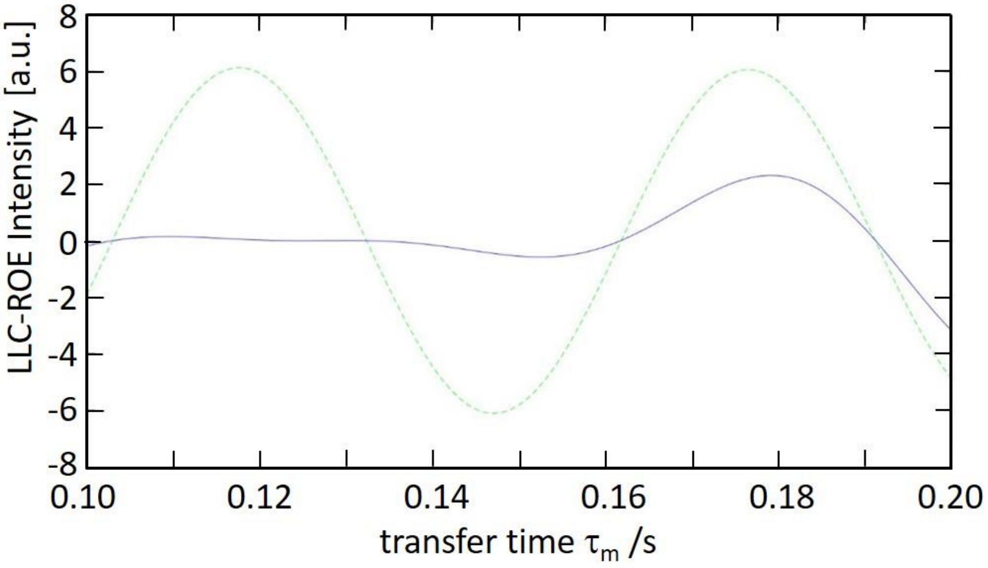

Figure S3. Expected rotating-frame transfer between LLC on Gly aliphatic protons (I,S) and an external proton spin K in the presence (blue) and absence (green) of small-frequency oscillations (here, a frequency of 2 Hz was considered) for spin K.

Matlab notebook for the calculation of the maximum LLC-ROE effect as function of rotation correlation time

```
% Maximum ROE transfer from Ix+Sx (REF) and LLC
% as function of rotational correlation time
clear all;
% close all;
% Spin system
% List of distances, angles and magnetizations
distlist = {2.5, 4, 6, 8};
anglelist = {0, 15, 30, 45};
maglist = {22.3, 11.7};
boollist = {1, 0}; %LLC or REF
for dist = 1:4
    for ang = 1:4
        close all;
        for magnet = 1:2
            for llc = 1:2
                disp([dist, ang, magnet, llc])
```

```
sys.magnet=maglist{magnet}; % 950 MHz = 22.3 T; 500 MHz = 11.7 T
sys.isotopes={'1H','1H','1H','1H'};
sys.output='hush';
dIS= 1.77; % Angstrom
dK = distlist{dist};  % Angstrom
dL = dK+2;  % Angstrom
theta=anglelist{ang}*pi/180;

% Zeeman interactions
inter.zeeman.scalar={-0.3 0.3 0.8 -0.8}; %Gly-49Hs & another K & L
inter.coupling.scalar{1,2}=17;
inter.coupling.scalar{3,4}=0;
inter.coupling.scalar{4,4}=0;
inter.coordinates={[-dIS/2,0,0],[dIS/2,0,0],[dK*sin(theta), dK*cos(theta),0], ...
   [dL*sin(theta), dL*cos(theta),0]};

%Basis formalism
bas.formalism='sphten-liouv';
bas.approximation='none';

maxLLC=[]; tau_C=[];
num_points = 25;

for j=logspace(-3,2,num_points) % Log scale for tauC = [1ps 100 ns]
   % Relaxation theory
   inter.relaxation={'redfield'};
   inter.equilibrium='zero';
   inter.rlx_keep='labframe';
   inter.tau_c={j*1e-9};   tau_C=[tau_C j*1e-9];

   % Spinach housekeeping
   spin_system=create(sys,inter);
   spin_system=basis(spin_system,bas);
   spin_system=assume(spin_system,'nmr');

   % Equilibrium density operator
   H_temp=hamiltonian(assume(spin_system,'labframe'),'left');
   rho_eq=equilibrium(spin_system,H_temp);

   % Operators and superoperators
   Ip=operator(spin_system,'L+',[1]);
   ISp=operator(spin_system,'L+',[1 2]);
   ISKp=operator(spin_system,'L+','1H');
   ISx=(ISp+ISp')/2; ISy=(ISp-ISp')/(2i);
   Ix=(Ip+Ip')/2; ISKx=(ISKp+ISKp')/2;
   H=hamiltonian(assume(spin_system,'nmr'),'comm');
   R=relaxation(spin_system);
   L=H+1i*R;


   %Build the component state
   Kp=state(spin_system,{'L+'},{3});
   Km=state(spin_system,{'L-'},{3});
   Kx=(Kp+Km)/2;

   % Run experiments
```

```
            amp_CW=4000; tau_CW=4/17; npoints=1000;
            rho1=step(spin_system,Ix,rho_eq,boollist{llc}*pi);
            rho2=step(spin_system,ISy,rho1,pi/2);

traj_ev1=evolution(spin_system,L+2*pi*amp_CW*ISKx,Kx,rho2,tau_CW/npoints,npoints,'observable');
            traj_ev2=evolution(spin_system,L-
2*pi*amp_CW*ISKx,Kx,rho2,tau_CW/npoints,npoints,'observable');
            traj_ev=traj_ev1+traj_ev2;

            maxLLC=[maxLLC max(abs(traj_ev))];
          end


          figure(1)
          semilogx(logspace(-3,2,num_points),maxLLC); hold on;
          plt=Plot();
          plt.LineWidth = [2];
          plt.BoxDim = [5 4];
          plt.LineStyle = {'-','-','--','--'};
          plt.Colors= {[0, 0, 0],[1, 0, 0],[0, 0, 0],[1, 0, 0]};
          plt.XLabel = '\tau_{C} [ns]';
          plt.YLabel = 'Maximum ROE transfer [a.u.]';
          plt.Legend = {'from I_{x}+S_{x} (B_{0} = 22.3 T)','from LLC (B_{0} = 22.3 T)'...
            'from I_{x}+S_{x} (B_{0} = 11.7 T)','from LLC (B_{0} = 11.7 T)'};
          plt.LegendLoc = 'NorthWest';
          plt.XScale = 'log';     % 'linear' or 'log'
          % plt.XGrid = 'on';       % 'on' or 'off'
        end
      end
      saveas(figure(1), strcat("a", string(anglelist{ang}), "d", string(distlist{dist}), '.png'));
      close all;
    end
end
```

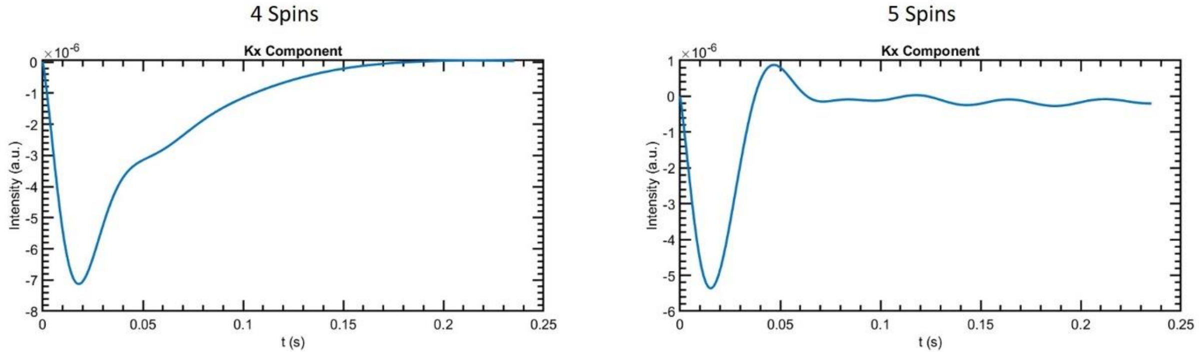

Figure S4. Calculation of LLC-ROE transfer from the 2-spin source to a 2-spin-system neighbour (i.e., 4-spin system, left) and a 3-spin-system neighbor (i.e. 5-sppin system, right); The interaction distance and angle (as in Fig. 1E) were chosen r = 5.7 Å and φ = 60°. Couplings within the neighbor spin system (K,M,N) were set to J(K,M)=8 Hz, J(K,N)=10 Hz,, J(M,N)=4 Hz.

Matlab code for 5-spin simulation of LLC transfer

```
clear all;
close all;
relaxation_spin = true;
five_spins = true;
proton_frequency = 500; %MHz
    dIS = 1.7561738;
    inter.zeeman.scalar = {-0.3 0.3 3.320};
    dK = 5.4548;
    theta1 = 0.350;
    % Spin system
sys.magnet = proton_frequency / 42.58;
sys.isotopes = {'1H', '1H', '1H'};
dL = dK + 1.5; % Angstrom
theta2 = theta1;
% Zeeman interactions
inter.coupling.scalar{1, 2} = 17;
inter.coupling.scalar{3, 3} = 0;
inter.coordinates = {[-dIS / 2, 0, 0], [dIS / 2, 0, 0], ...
            [dK * cos(theta1), dK * sin(theta1), 0]};

% Add the spin for relaxation if desired
if five_spins
  sys.isotopes = [sys.isotopes, '1H', '1H'];
```

```
    inter.zeeman.scalar = [inter.zeeman.scalar, -1 * inter.zeeman.scalar(end), inter.zeeman.scalar(end)];
    inter.coupling.scalar{5, 5} = 0;
    inter.coupling.scalar{3, 4} = 10;
    inter.coupling.scalar{3, 5} = 8;
    inter.coupling.scalar{4, 5} = 4;

    inter.coordinates = [inter.coordinates, [dL * cos(theta2), dL * sin(theta2), 0], [dL * cos(theta2) + 1.5, dL * sin(theta2) + 1.5, 0]];

elseif relaxation_spin

    sys.isotopes = [sys.isotopes, '1H'];
    inter.zeeman.scalar = [inter.zeeman.scalar, -1 * inter.zeeman.scalar(end)];
    inter.coupling.scalar{4, 4} = 0;
    inter.coupling.scalar{3, 4} = 2;
    inter.coordinates = [inter.coordinates, [dL * cos(theta2), dL * sin(theta2), 0]];

end

% Basis formalism
bas.formalism = 'sphten-liouv';
bas.approximation = 'none';
% Relaxation theory
inter.relaxation = {'redfield'};
inter.equilibrium = 'dibari';
```

```
inter.temperature = 298;
inter.rlx_keep = 'secular';
inter.tau_c = {6 * 1e-09};

% Spinach housekeeping
spin_system = create(sys, inter);
spin_system = basis(spin_system, bas);
% Equilibrium density operator
H_temp = hamiltonian(assume(spin_system, 'labframe'), 'left');
rho_eq = equilibrium(spin_system, H_temp);
% Operators and superoperators
Ip = operator(spin_system, 'L+', [1]);
ISp = operator(spin_system, 'L+', [1 2]);
ISKp = operator(spin_system, 'L+', '1H');
ISx = (ISp + ISp') / 2; ISy = (ISp - ISp') / (2i);
Ix = (Ip + Ip') / 2; ISKx = (ISKp + ISKp') / 2;
H = hamiltonian(assume(spin_system, 'nmr'), 'comm');
R = relaxation(spin_system);
L = H + 1i * R;
amp_CW = 4000; tau_CW = 4/17; npoints = 1024;
rho1 = step(spin_system, Ix, rho_eq, pi);
rho = step(spin_system, ISy, rho1, pi / 2);
% rho=-rho_eq;
% Projections
LLC = (state(spin_system, 'L+', [1]) + state(spin_system, 'L-', [1])) / 2 - ...
```

```
    (state(spin_system, 'L+', [2]) + state(spin_system, 'L-', [2])) / 2;
Kx = (state(spin_system, 'L+', [3]) + state(spin_system, 'L-', [3])) / (2);
Ky = (state(spin_system, 'L+', [3]) - state(spin_system, 'L-', [3])) / (2i);
Kz = state(spin_system, 'Lz', [3]);

traj_plus = evolution(spin_system, L + 2 * pi * amp_CW * ISKx, [], rho, tau_CW /
npoints, npoints - 1, 'trajectory');
    traj = traj_plus;
time = linspace(0, tau_CW, size(traj, 2));

% figure(); hold on;
% subplot(4, 1, 1); hold on;
% plot(time, LLC' * traj);
% legend('LLC');

% subplot(4, 1, 2); hold on;
% plot(time, Kx' * traj);
% legend('Kx');

% subplot(4, 1, 3); hold on;
% plot(time, Ky' * traj);
% legend('Ky');

% subplot(4, 1, 4); hold on;
% plot(time, Kz' * traj);
% legend('Kz');
```

```
figure(); hold on;
plot(time, LLC' * traj, "LineWidth", 2.2);
plt = Plot();
plt.Title = "LLC Intensity Evolution"
plt.LineWidth = 2;
plt.XLabel = 't (s)'; % xlabel
plt.YLabel = 'Intensity (a.u.)'; %ylabel

figure(); hold on;
plot(time, Kx' * traj);
plt = Plot();
plt.LineWidth = 2;
plt.Title = "Kx Component"
plt.XLabel = 't (s)'; % xlabel
plt.YLabel = 'Intensity (a.u.)'; %ylabel
```

3.3 Supplementary Note 3.3: Build-up in NOE vs ROE and ROE_LLC spectroscopy

The ratio of the enhancements for NOE and ROE transfer for a large molecule ($\omega_0\tau_c \gg 0$), is

$$\frac{\eta_{NOE}}{\eta_{ROE}} = 1.25$$

The pertaining calculations [8,9] are detailed here.

In a homonuclear system:

$$\eta_{NOE} = \frac{\sigma_{NOE}}{R_{1I}}\frac{\gamma_I}{\gamma_S} = \frac{\sigma_{NOE}}{R_{1I}}$$

In the slow-diffusion limit ($\omega_0\tau_c \gg 0$), this value becomes:

$$\eta_{NOE} = -1$$

The value of R_{1I} can be computed as:

$$R_{1I} = \frac{d_{00}}{4}[J(\omega_I - \omega_S) + 3J(\omega_I) + 6J(\omega_I + \omega_S)]$$

where

$$d_{00} = \left(\frac{u_0}{4\pi}\right)^2 \frac{\hbar^2\gamma_I^2\gamma_S^2}{r_{IS}^6}$$

Regarding the ROE, we have in the slow-diffusion limit:

$$\sigma_{ROE} = -2\sigma_{NOE}$$

The transverse relaxation rate constant in the rotating frame is:

$$R_{2I} = \frac{d_{00}}{8}[4J(0) + J(\omega_I - \omega_S) + 3J(\omega_I) + 6J(\omega_S) + 6J(\omega_I + \omega_S)]$$

In the same slow-tumbling limit, this becomes:

$$R_{2I} = \frac{5d_{00}}{8}J(0)$$

Therefore,

$$\frac{\eta_{IS}^{NOE}}{\eta_{IS}^{ROE}}=1.25$$

Given that we have experimentally obtained values of $\eta^{ROE\ LLC}$ up to two times larger than "standard" η^{ROE} measurements, we can reasonably expect this method to also provide an improvement over the NOE measurement.

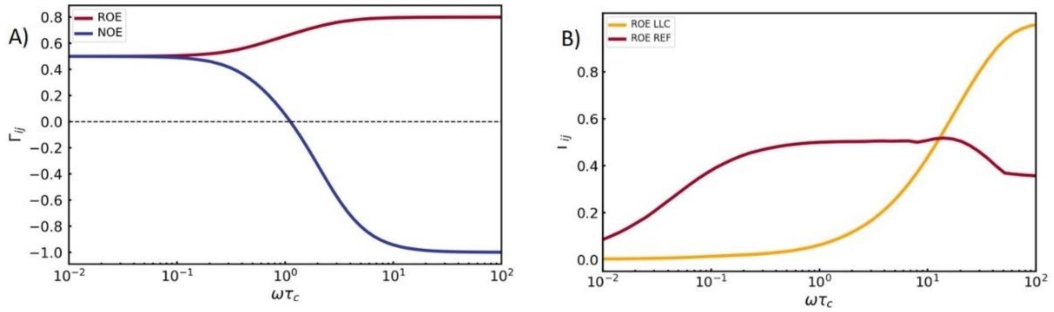

Figure S5 A) (two-spins systems) Comparison of Overhauser transfer Γ_{ij} in 2-spin systems according to calculations above, plotted as in H. Desvaux, P. Berthault, N. Birlirakis, M. Goldman, "Off-Resonance ROESY for the study of dynamic processes", J. Magn. Reson. Ser. A. 108 (1994) 219 229; B) (three-spins systems) Comparison of ROE-LLC and standard ROE transfer between a two-spin source and a third spin. (Spinach simulation).

4. Supplementary Note 4: 2D ROE LLC experiments and spatial neighbor assignments

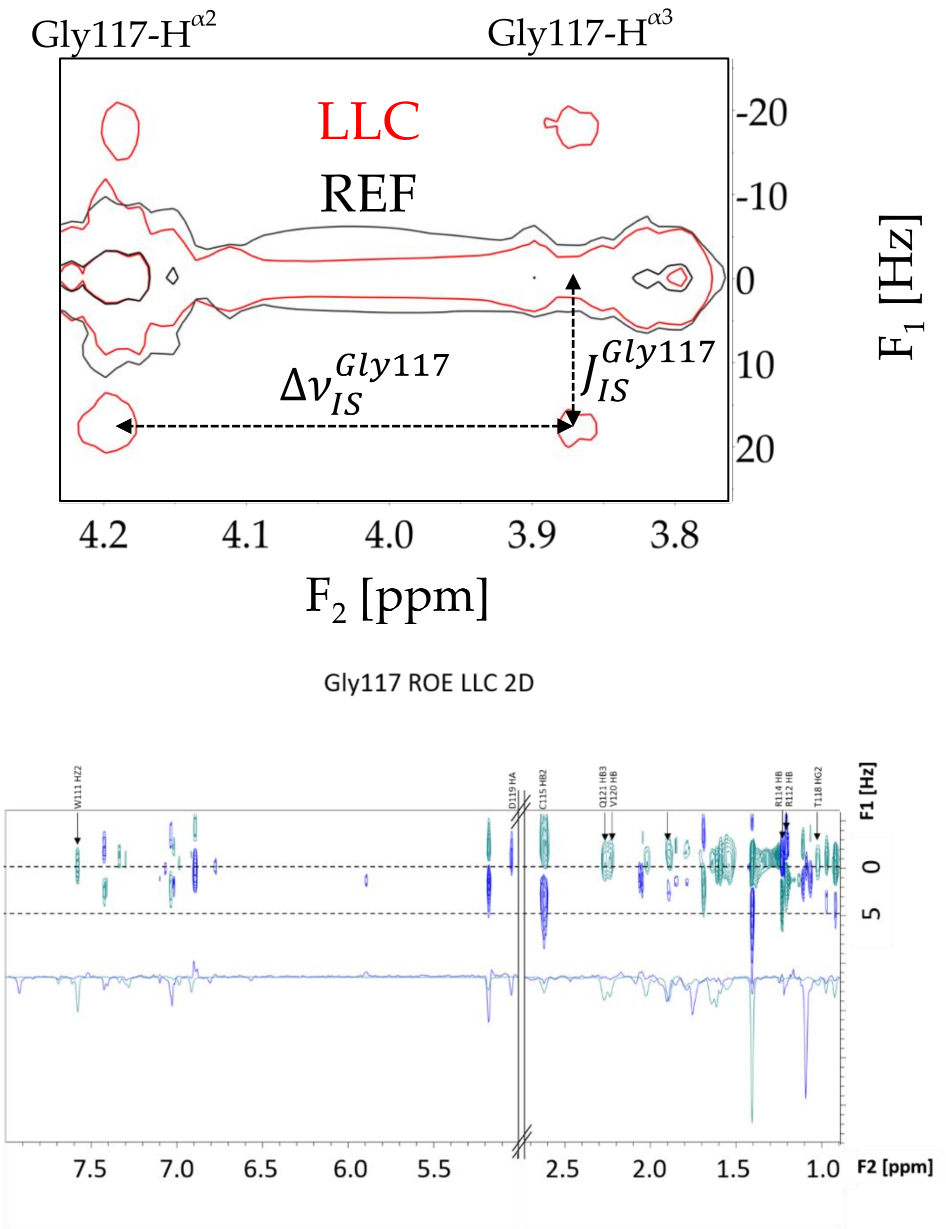

Figure S6 (top) Source LLC for Gly-117 in 2D spectroscopy. (bottom) ROE LLC 2D spectrum (recorded with pulse sequence from Fig 1C) superimposed with slices from ROESY 2D spectrum for Gly117 (in green – 1D slice at $H^{\alpha 2}$ frequency, in blue – 1D slice at $H^{\alpha 3}$ frequency). Spatial neighbors within a 10 Å radius have been assigned based on the 9LYZ PDB structure. Strong signals that could not be assigned with certainty are likely to originate from: Trp111(HD1, HH2, HE3) in the 6.5-7.5 ppm region, respectively T118, V120 HG in the 1-2 ppm region.

5. Supplementary Note 5: Lysozyme interactions with trisaccharide NAG-NAM-NAG

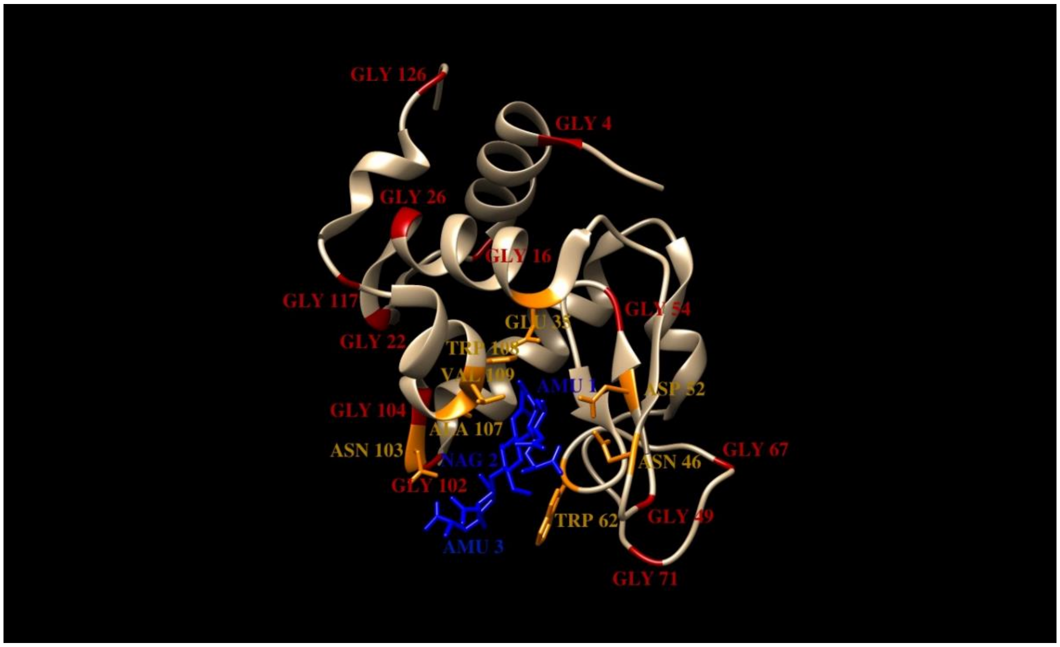

Figure S7. Interaction of trisaccharide NAM-NAG-NAM (blue) with Lysozyme based on PDB structure ‘9LYZ’. Long-lived coherences excited on aliphatic protons of widespread Gly residues can sense biological interactions directly or via Overhauser-interaction mediated transfers (e.g., Gly49$H^{\alpha 2,3}$ interact with Asn46H^{α}, which is in direct contact with the ligand).

Supplementary References

[1] T. E. Bull, Relaxation in the Rotating Frame in Liquids, Progress in Nuclear Magnetic Resonance Spectroscopy 24, 377 (1992).
[2] C. Bengs and M. H. Levitt, SpinDynamica: Symbolic and Numerical Magnetic Resonance in a Mathematica Environment, Magnetic Resonance in Chemistry 56, 6 (2018).
[3] R. Sarkar, P. Ahuja, P. R. Vasos, A. Bornet, O. Wagnières, and G. Bodenhausen, Long-Lived Coherences for Line-Narrowing in High-Field NMR, Progress in Nuclear Magnetic Resonance Spectroscopy 59, 1 (2011).
[4] F. Teleanu, A. Topor, D. Serafin, A. Sadet, and P. R. Vasos, Rotating-Frame Overhauser Transfer via Long-Lived Coherences, Symmetry 13, 9 (2021).
[5] H. Geen and R. Freeman, Band-Selective Radiofrequency Pulses, Journal of Magnetic Resonance (1969) 93, 93 (1991).
[6] F. Teleanu, A. Lupulescu, and P. R. Vasos, Selective Excitation of Long-Lived Nuclear Spin States, J. Phys. Chem. Lett. 13, 6731 (2022).
[7] H. J. Hogben, M. Krzystyniak, G. T. P. Charnock, P. J. Hore, and I. Kuprov, Spinach – A Software Library for Simulation of Spin Dynamics in Large Spin Systems, Journal of Magnetic Resonance 208, 2 (2011).
[8] I. Solomon Relaxation Processes in a System of Two Spins, Phys. Rev. 99, 559 (1955)
[9] Cavanagh, J. et al, Protein NMR Spectroscopy, Second Edition, Principles and Practice, Academic Press (2006)
[10] H. Desvaux, P. Berthault, N. Birlirakis, M. Goldman, Off-Resonance ROESY for the study of dynamic processes, J. Magn. Reson. Ser. A. 108 (1994) 219–229
